# Monitoring of pre-frontal oxygen status in helicopter pilots using near-infrared spectrophotometers

**DOI:** 10.1186/1476-5918-7-10

**Published:** 2008-07-11

**Authors:** Azusa Kikukawa, Asao Kobayashi, Yoshinori Miyamoto

**Affiliations:** 1 Aeromedical Laboratory, Japan Air Self-Defense Force, Tachikawa, Tokyo, Japan

## Abstract

**Background:**

There are few in-flight studies of cognition-related cerebral oxygen status in helicopter pilots.

**Methods:**

Four male helicopter pilots volunteered for nine sorties during visual flight in a BK117 and UH-60J. The pilots' pre-frontal oxy-hemoglobin (O_2_Hb) and deoxy-hemoglobin (HHb) concentration were continuously monitored from the right/left sections of the forehead using near-infrared spectrophotometers with a consideration of motion artifacts.

**Results:**

The concentration of O_2_Hb progressively increased (13.98 μmol•L^-1 ^as a maximum increased concentration) in both the right/left sections of the forehead from the basal level during the heightened cognitive demand of helicopter flight. There was comparatively little change (4.32 μmol•L^-1 ^as a maximum increased concentration) in HHb concentration during measurement of helicopter flight. HHb changes were apparently not affected by a heightened cognitive demand of helicopter pilots.

**Conclusion:**

These results demonstrate that near-infrared spectroscopy, especially O_2_Hb measurements, provides a sensitive method for the monitoring of cognitive demand (maneuvers) in helicopter pilots.

## Background

Near-infrared spectroscopy (NIRS) has become an acceptable, non-invasive method for the quantitative measurement of pre-frontal oxygen status (PFOS; pre-frontal oxy-hemoglobin concentration change), including cognition-related status [1-4], and previous studies have demonstrated it to be a reliable and sensitive method in aviation environments [5-13]. A better understanding of PFOS in terms of cortical signals during actual flight is needed to increase its applicability to flight safety. Moreover, the rapid, accurate, and continuous monitoring of pilot state and/or workload is highly desirable in modern aircraft systems [14]. Recently, NIRS has been successfully used to measure the in-flight cerebral oxygen status of F-15 fighter pilots during aerial gunnery training [8] and during air-to-air combat maneuvering [11]. Technological advances and a heavy workload have resulted in increasing operational demands on both military and civilian helicopter pilot, so it is critical to understand the pilots' cerebral oxygen status during helicopter flight missions. However, NIRS measurements can be compromised by movements of the head, especially for real world applications, where the movement of the head cannot be restricted, as in studies involving pilots, children, etc. [15]. Accordingly, little is known at present about the PFOS (as a cerebral oxygen status) of helicopter pilots during actual flight. In helicopter flight, one study investigated the use of NIRS for critical patients in air medical transport, but this study did not examine the PFOS of the helicopter pilot [9]. The aim of this study was to examine cognition-related PFOS in helicopter pilots during actual flight using NIRS with a consideration of motion artifacts.

## Methods

Four male helicopter pilots in the Japan Air Self-Defense Force (JASDF) volunteered to perform nine sorties with an in-flight examination BK117 and UH-60J single rotor helicopters (Table 1). All the pilots had passed an extensive physical examination within the past 12 months and were healthy. The procedures for this study were approved by the Aeromedical Laboratory Committee in JASDF. All subjects were fully briefed on the scope of the experiment, and written informed consent was obtained from all subjects before the experiment.

**Table 1:** Pilot Characteristics.

***Pilot***	***Age (yr)***	***Height (cm)***	***Weight (kg)***	***Flying Hours (hr)***	***Helicopter***	***Number of Sorties***
*A*	49	173	67	4735	BK117	4
*B*	46	164	57	5715	BK117	3
*C*	41	168	71	4112	UH-60J	1
*D*	34	168	64	1899	UH-60J	1

### Laboratory measurements

To determine the effects of head movement on NIRS measurement, healthy subjects were also studied under laboratory conditions. The healthy, right handed subjects (12 pilots and 8 non-pilots) volunteered for this study. Age ranged from 23 to 43 years with an average of 30.8 years. In the laboratory measurements, the subjects did not wear a helmet, and BK117 and UH-60J helicopter pilots were not included. The intake of caffeinated drinks and smoking were prohibited from the time of rising in the morning to the time of the end of the measurement to prevent any effect of these substances. For the laboratory participants, measurements were conducted in a quiet laboratory where the room temperature was maintained between 20 and 27°C. The effects of a 45° downward head movement on the O_2_Hb/HHb concentration as well as those of a 45° head upward and 80° right/left movements were examined. The duration of the head movements (up, down, left, and right) was 10 seconds. Baseline measurements were obtained with subjects sitting on a chair. The concentration changes in O_2_Hb/HHb were tested for the comparison between the right and left forehead using paired t-test. The concentration changes in O_2_Hb/HHb during head movement compared to baseline were tested using Wilcoxon Signed Rank Test. Statistical significance was considered to be present at p < 0.05.

### Flight mission measurements

The pilots' cerebral hemoglobin oxygenation status was continuously monitored from the right and left portions of the forehead using two near-infrared spectrophotometers (NIRO-300G, Hamamatsu Photonics K.K., Japan). The detailed principles of the NIRS technique are described elsewhere [16-19]. The instrument in this study has been described previously [8,11], but essentially, intracranial concentration changes of oxyhemoglobin (O_2_Hb) and deoxy-hemoglobin (HHb) can be measured using the NIRO-300G. Gy (leftward/rightward acceleration force), and Gz (headward/footward acceleration force) were also monitored simultaneously during the flight using low-capacity acceleration transducers (AS10GB, Kyowa Electronic Instruments Co., LTD., Tokyo, Japan).

Head and whole body movements of the pilots were determined, and helicopter situational data were also recorded throughout the NIRS measurements by a video recorder. The video data was captured on a videotape recorder (Video Hi8 Handycam, CCD-TR 3000, Sony Co., Japan) which was installed into the cockpit in the left front of the pilot area. Additionally, voice data was recorded by a microcassette-recorder (M-950, Sony Co., Japan) to monitor the events during the flight missions.

Before each data collection flight, just prior to the experiments, the left and right probes were taped to either side of the midline on the subject's forehead. Thus two probes were placed on the right and left side of forehead at approximately 2 centimeters above the eyebrows and approximately 3 centimeters from the midline. This position avoided false readings related to the frontal sinus. Moreover, probes were placed following a procedure that ensured a reproducible, uniform separation distance of 4.0 cm while avoiding the region of the temporal muscles. The two NIRO-300Gs were synchronized to each other in time and to accelerometers which monitored G-levels. A researcher rode in the back seat to observe pilots reactions, as well as to monitor and to input the events. These data were noted in the survey. The phases of flight investigated included at rest in the cockpit, taxiing, takeoff, level flight, approach to the top of the mountain, and landing. The actual helicopter flight was approximately 2.0 hours between 0830~1730. The BK117 is not equipped with an automatic flight control system (AFCS). Otherwise, the UH-60J aircraft is equipped with an AFCS that enhances the hover stability and handling qualities. In this study, all NIRS measurements were performed in visual flight without an AFCS. UH-60J pilots wore helmets during the flight missions. On the other hand, BK 117 pilots did not wear helmets for the experimental measurements.

## Results

### Laboratory measurements

**Figure 1:**
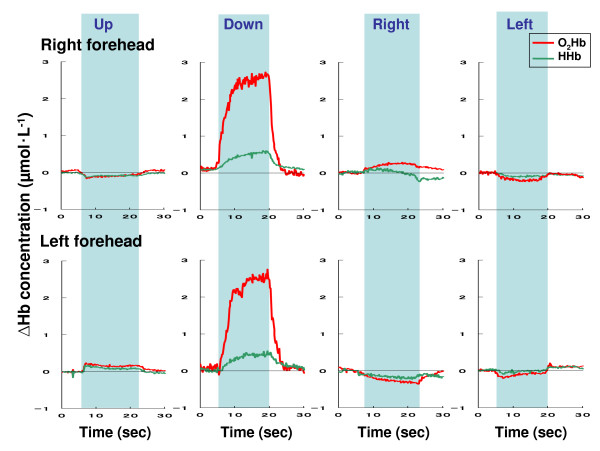
Representative samples of oxy-hemoglobin (O_2_Hb) and deoxy-hemoglobin (HHb) changes in right and left frontal regions obtained from a subject during the head movement in laboratory measurement.

Figure 1 shows representative O_2_Hb and HHb changes in the right and left frontal regions obtained from a subject during head movement in the course of laboratory measurement with NIRS. In all measurements, the O_2_Hb concentrations increased or decreased immediately after the head movement, and then the O_2_Hb concentration also immediately returned to the baseline levels at the initial (horizontal) position. As a result, the time intervals of the cerebral O_2_Hb changes are considered to be sufficient to stabilize the O_2_Hb response, which has been shown to occur within 3 seconds of the rise and fall timepoints. Table 2 shows the concentration changes of O_2_Hb and HHb in the right and left forehead from baseline during head movement. During laboratory experiments, there was little change in HHb. O_2_Hb and HHb concentration changes did not differ significantly at baseline during upward, right and left head movements, whereas in the case of downwards head movement, the O_2_Hb concentrations significantly changed. Otherwise, the O_2_Hb and HHb concentrations were not significantly changed between the right and left forehead during laboratory measurements. Thus the concentration of O_2_Hb and HHb in the right/left forehead ranged from -1.527 ~ +0.531 μmol•L^-1 ^and -1.024 ~ +0.620 μmol L^-1 ^in comparison to each baseline. In downward head movement, the concentration of O_2_Hb and HHb in the right/left forehead ranged from +2.769 ~ +3.205 μmol•L^-1 ^and +0.108 ~ +1.491 μmol L^-1 ^as compared to each baseline.

**Table 2:** The concentration changes of oxy-hemoglobin (O_2_Hb) and deoxy-hemoglobin (HHb) from baseline in laboratory measurements taken during head movements.

	***Concentration changes of hemoglobin (average ± standard deviation) μmol•L***^*-1*^			
	
***Head movement***	***O***_2_***Hb***		***HHb***	
		
	***Left forehead***	***Right forehead***	***Left forehead***	***Right forehead***
*Up*	-0.211 ± 1.620	-0.687 ± 1.566	-0.060 ± 0.381	-0.181 ± 0.403
*Down*	2.281 ± 2.529**	3.110 ± 2.580**	0.713 ± 0.559**	0.782 ± 0.568**
*Right*	-0.338 ± 0.769	-0.002 ± 1.144	-0.088 ± 0.219	0.005 ± 0.313
*Left*	-0.531 ± 1.231	-0.753 ± 1.792	-0.115 ± 0.315	-0.226 ± 0.458

** Significantly change from baseline level.

### Flight mission measurements

**Figure 2:**
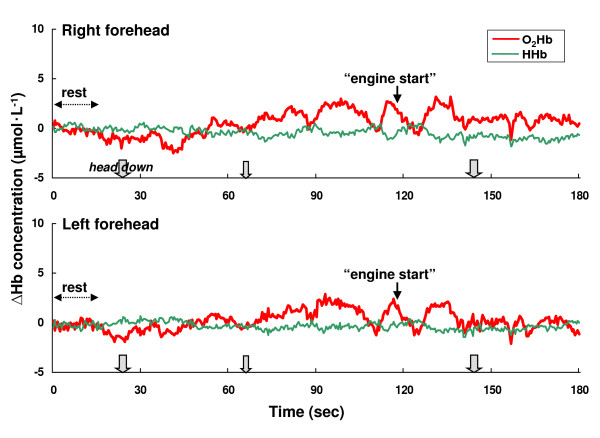
**An example of the changes in oxy-hemoglobin (O_2_Hb) and deoxy-hemoglobin (HHb) in right and left frontal regions during BK117 flight**. The dotted arrow indicates the flight situation. The solid arrow indicates a call by the pilot. The outline arrow indicates the downward head movement of the pilot (bold: 2~3 seconds, thin: 1~2 seconds).

**Figure 3:**
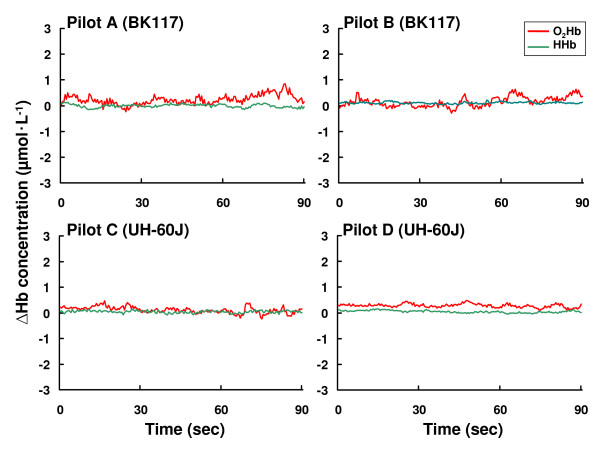
The concentration change of oxy-hemoglobin (O_2_Hb) and deoxy-hemoglobin (HHb) during level flight in each pilot.

In this study, similar changes in PFOS with NIRS were observed in all pilots during the same flight tasks. Figure 2 shows an example of the changes in concentration of O_2_Hb and HHb in the bilateral regions during the BK117 flight. There was no difference in the trend in PFOS changes between the right and left frontal regions of the subjects. Thus, in this study, we present data obtained from the right frontal region as a typical example of NIRO-300Gs data. Figure 3 shows the changes of O_2_Hb and HHb during level flight for each subject where the concentration changes of O_2_Hb and HHb are maintained at a lower level (< 0.04 μmol•L^-1^). As shown in Figure 3, the effects of vibration in the helicopter had no evident effect on the NIRS signals.

**Figure 4:**
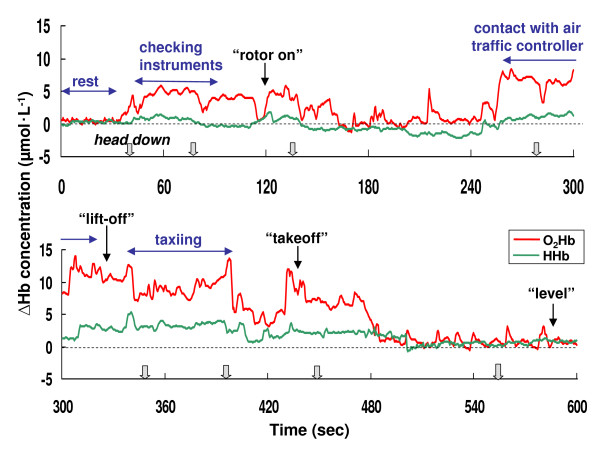
**The concentration changes of oxy-hemoglobin (O_2_Hb) and deoxy-hemoglobin (HHb) during the takeoff period in pilot A with the BK117**. The dotted arrow indicates the flight situation. The solid arrow indicates a call by the pilot. The outline arrow indicates the downward head movement (1~2 seconds) of the pilot.

**Figure 5:**
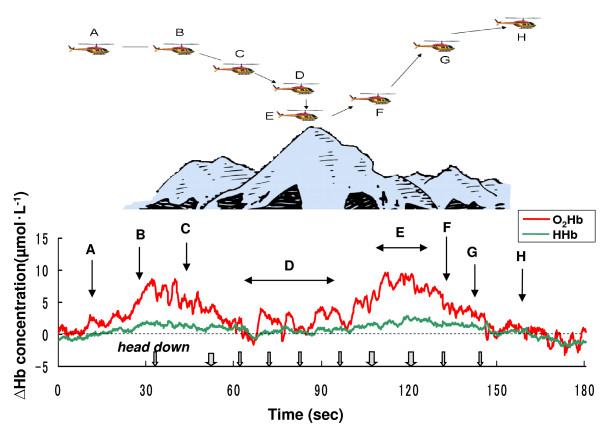
**The oxy-hemoglobin (O_2_Hb) and deoxy-hemoglobin (HHb) changes and position of the aircraft during an approach to the top of the mountain in pilot A with the BK117**. Upper part: flight course and aircraft position, lower part: the NIRS data. The outline arrow indicates the downward head movement of the pilot (bold: 2~3 seconds, thin: 1~2 seconds).

**Figure 6:**
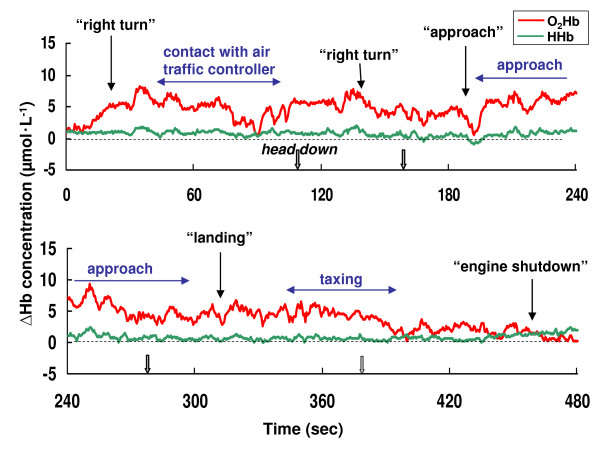
**The concentration changes of oxy-hemoglobin (O_2_Hb) and deoxy-hemoglobin (HHb) during landing period in pilot B with the BK117**. Dotted arrow indicates the flight situation. Solid arrow indicates the call of pilot. Outline arrow indicates head down movement (1~2 seconds) of the pilot.

Representative samples of the NIRO-300Gs data from the subjects during the flight missions are presented in Figures 4 through 6. Figure 4 displays examples of the PFOS, presented as the O_2_Hb and HHb changes observed during the takeoff period in the BK117. The concentration of O_2_Hb during the activities of checking instruments, contact with the air traffic controller, and taxiing progressively increased from the basal level (13.98 μmol•L^-1 ^as a maximum increased concentration). The O_2_Hb concentration also increased after the command "rotor on" and immediately before "takeoff" (12.03 μmol•L^-1 ^as a maximum increased concentration). After pilots achieved level flight, the concentration of O_2_Hb returned to the baseline level. Figure 5 and Figure 6 also show an example of the PFOS during an approach to the top of a mountain and landing mission in a BK117 pilot, respectively. The concentration of O_2_Hb during these flights with cognition-related tasks also increased from the basal level (9.29 μmol•L^-1 ^as a maximum increased concentration). Otherwise, there was comparatively little change in HHb concentration from the basal level during the flight mission measurements (4.32 μmol•L^-1 ^as a maximum increased concentration).

The video data and researcher observation showed that the period of the downward head movement was within a 1~3 second range per movement, and then immediately returned to the normal position. The Gz values during the flight study were within the range of 0.8 to 1.4 G.

## Discussion

The purposes of this study were to: 1) examine the PFOS of helicopter pilots during actual flight using NIRS measurements; and 2) determine whether cognition-related O_2_Hb changes could be detected during actual flight. It has previously been shown that NIRS is able to detect changes in blood oxygenation caused by cognitive demands [1-4,20-22]. Moreover, NIRS provides a relatively inexpensive and robust monitor for cortical activation, a metric important for determining the cognitive response efficiency for the instantaneous gross mental activity level [23]. Hoshi et al. also demonstrated that an increase in O_2_Hb showed a positive correlation with task difficulty [24].

However, motion artifacts in NIRS studies are a potentially serious problem for real life applications [15]. Motion artifacts such as arise with head movement can cause the blood to move toward (or away from) the area that is being monitored, increasing (or decreasing) the amount of oxygen, hence resulting in an increase (or decrease) in the collected data [15]. In this study, accordingly, the effects of head movement on NIRS measurement in healthy subjects were studied under laboratory conditions. The laboratory study showed that the concentration of O_2_Hb changed in the range of -1.53 ~ +0.53 μmol•L^-1 ^from baseline during upward and right/left head movements (Table 2). Thus, there was little change in the O_2_Hb concentration, so it is considered that these head movements (upward and right/left) employed in this study will have no affect on PFOS measurement during actual helicopter flight as cognition-related changes. In the laboratory study, however, the concentration of O_2_Hb was increased to +3.11 μmol•L^-1 ^during downward head movement (Table 2). Thus, it was hypothesized that the concentration of O_2_Hb with downward head movements would potentially significantly affect the flight study. Therefore head and body movements of the pilots were monitored with a video recorder during the NIRS measurements, except for the case of the artifact caused by the downward head movement of the subjects. Additionally, a researcher rode in the back seat to observe the pilot reactions, and to monitor the downward head movements. The data showed that the period of the downward head movement in helicopter pilots was within a 1~3 second range per head movement (Figure 2, Figure 4, Figure 5, and Figure 6), and then the head position immediately returned to the initial (horizontal) position. As noted above, it was hypothesized that the downward head movement would lead to a motion artifact in the NIRS studies during actual helicopter flight. Although the results of the downward head movement in the laboratory study lead to the conclusion that there could be an abrupt O_2_Hb increase, this was not observed during the actual flight study (Figure 2, Figure 4, Figure 5, and Figure 6). There is a possibility that the increase of O_2_Hb concentration was a result of cognitive demand (related to task difficulty) during the flight tasks which masked the increase in the O_2_Hb levels as an effect of head down movement. The total period of the downward head movement during the flight was observed occurred within 10 seconds to about 10 minutes of flight, which is a relatively long period (Figure 4). Due to the fact that the ratio of the period (downward head movement/~10 minutes flight) is approximately one-sixtieth, it was thought that this movement would be negligible as a motion artifact during the relatively long period of the helicopter flight. However, consideration of the PFOS data from downward head movement may still be important during the relatively short period (i.e., one or two minutes) of the in-flight PFOS study.

On the other hand, the possibility must also be considered that the pilot helmet can change the pressure on the NIRS probes. However, there is no difference in the NIRS measurements between the BK117 pilots without helmets and UH-60J with helmets (Figure 3). In this study, whole body movement, which is mainly comprised of arm and leg movements, resulted in unchanged O_2_Hb levels and did not affect the monitoring of the PFOS in helicopter pilots during level flight (Figure 3). Recently, Jayakar et al. reported that mouth movement increased regional cerebral oxygenation values in normal adult subjects while in the supine position [25]. However, as shown in Figure 3 (as the pilot talked to the researcher during level flight), increases of O_2_Hb concentrations due to mouth movement with verbalization were not detected. This may be due to differences in the measurement conditions, such as a difference in the subjects' position and/or equipment sensitivity.

Gy and Gz were monitored simultaneously during the flight. Gz values during the flight study were within the range of 0.8 to 1.4, and there was little change in Gy. The fact is that we did not observe O_2_Hb changes rerated to the Gy and Gz. We conclude that these factors thus did not affect the O_2_Hb changes.

Previous research has suggested that the HHb signal is a reflection of O_2 _extraction [26]. In general, total hemoglobin concentration, which is a measure for blood volume, is defined as the sum of O_2_Hb and HHb concentration. In this study, there was comparatively little change in HHb during the measurements of laboratory and flight missions (Figure 1~6, Table 2). These results for HHb might be caused by the fact that the changes in HHb are often much smaller than in O_2_Hb. Then, HHb changes were apparently not affected by a heightened cognitive demand of helicopter flights.

In performing the flight measurements, we were not able to successfully measure the absolute tissue hemoglobin saturation (tissue oxygen index) [27]. Previously, fMRI-NIRS or PET NIRS simultaneous measurement studies have reported that O_2_Hb is strongly correlated with the fMRI signal and rCBF [28-30]. A number of NIRS measurements of cerebral O_2_Hb have demonstration an increased (or decreased) pattern induced by various cognitive tasks [31-34].

Taken together then, the measurement of O_2_Hb changes that we observed in NIRS comprises a sensitive method for investigating changes in PFOS in helicopter pilots during flight missions. However, it should be mentioned that the effect of the increased O_2_Hb concentration measured in this study is a compensatory mechanism of brain tissue due to the initial deoxygenation caused by the task [35].

In this study, cognitive demands (tasks) during mission flights seem to have had a markedly stronger influence on increases in frontal O_2_Hb concentrations than other flight missions, such as those in level flight. Thus, one of the heightened risks of aircraft accidents is probably the reasons for the increase in the cognitive demand (task) component during takeoff, approach-and-landing and strenuous missions. In this study, the increase in concentration of O_2_Hb from the basal level during taxi-out/takeoff strongly suggests the O_2_Hb concentration is associated with cognitive demand (maneuvers) in helicopter pilots, and this O_2_Hb increase in pilots was detectable via NIRS with a monitoring of downward head down movement to eliminate artifacts (Figure 4). In general, it is very difficult for a helicopter pilot to carry out an approach to the top of a mountain. As shown in Figure 5, the concentration of O_2_Hb during strenuous missions such as an approach to the top of a mountain also increased from the basal level (see Figure 5, aircraft position from B to D, and during E).

As described above, our in-flight measurements indicate that O_2_Hb concentration increases as a function of heightened flying demands. Level flight decreases cognitive demands and, therefore, the concentration of O_2_Hb is maintained at a lower level (Figure 3). A previous study reported the right frontal cortex dominates sympathetic activity during stress-inducing mental tasks, including emotional stimuli. However, the findings obtained by various different investigations are contradictory. In several neuroimaging studies, no clear lateralization or functional role of the frontal cortex in automatic nervous system regulation during stress-inducing mental tasks was observed [36]. As shown in Figure 2, a similar pattern was detected in PFOS changes between right and left frontal regions of the subjects. The results support the hypothesis that there is no clear lateralization or functional role of the frontal cortex in PFOS during helicopter flight missions.

## Conclusion

The present study is one of the first to examine the relationship between actual flight and PFOS in helicopter pilots. The observed increase of O_2_Hb appear primarily to be a result of cognitive demand during the flight tasks. The results clearly demonstrate that NIRS provides a sensitive method for the monitoring of cognitive demand in helicopter pilots. Moreover, NIRS measurement with the use of a video-recorder as an artifact eliminator, such as a head down movement, can be used as a monitor of the PFOS during actual helicopter flight.

## Competing interests

The authors declare that they have no competing interests.

## Authors' contributions

AK participated in the sequence alignment and drafts the manuscript. AK participated in the design of the study. YM conceived of the study and in its design and coordination and helped to draft the manuscript. All authors read and approved the final manuscript.
